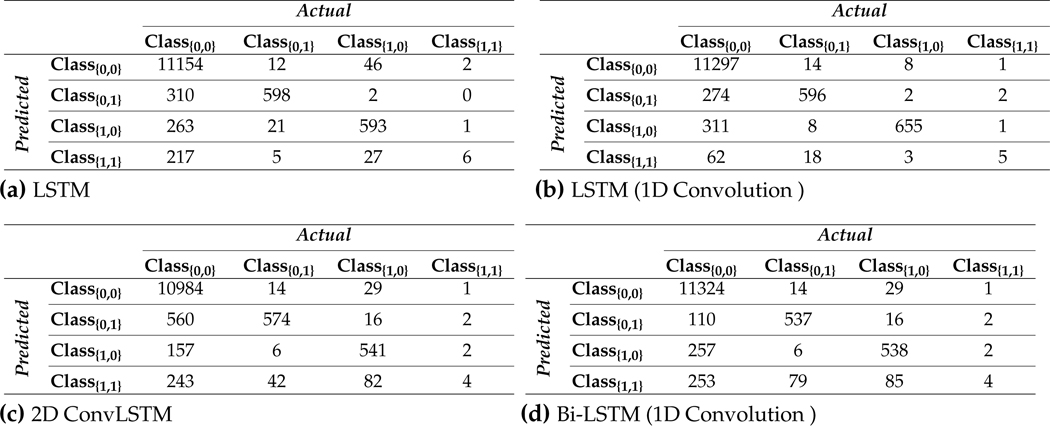# Meal and Physical Activity Detection from Free-living Data for Discovering Disturbance Patterns to Glucose Levels in People with Diabetes

**DOI:** 10.3390/biomedinformatics2020019

**Published:** 2022-06-01

**Authors:** Mohammad Reza Askari, Mudassir Rashid, Xiaoyu Sun, Mert Sevil, Andrew Shahidehpour, Keigo Kawaji, Ali Cinar

**Affiliations:** 1 Department of Chemical and Biological Engineering, Illinois Institute of Technology, Chicago, Illinois 60616, United States; 2 Department of Biomedical Engineering, Illinois Institute of Technology, Chicago, Illinois 60616, United States

**Keywords:** Recurrent Neural Networks, Event Detection, Data Preprocessing, Outlier Removal, Type 1 Diabetes

## Abstract

**Objective:**

Interpretation of time series data collected in free-living has gained importance in chronic disease management. Some data are collected objectively from sensors and some are estimated and entered by the individual. In type 1 diabetes (T1D), blood glucose concentration (BGC) data measured by continuous glucose monitoring (CGM) systems and insulin doses administered can be used to detect the occurrences of meals and physical activities and generate the personal daily living patterns for use in automated insulin delivery (AID).

**Methods:**

Two challenges in time-series data collected in daily living are addressed: data quality improvement and detection of unannounced disturbances to BGC. CGM data have missing values for varying periods of time and outliers. People may neglect reporting their meal and physical activity information. In this work, novel methods for preprocessing real-world data collected from people with T1D and detection of meal and exercise events are presented. Four recurrent neural network (RNN) models are investigated to detect the occurrences of meals and physical activities disjointly or concurrently.

**Results:**

RNNs with long short-term memory (LSTM) with 1D convolution layers and bidirectional LSTM with 1D convolution layers have average accuracy scores of 92.32% and 92.29%, and outper-form other RNN models. The F1 scores for each individual range from 96.06% to 91.41% for these two RNNs.

**Conclusions:**

RNNs with LSTM and 1D convolution layers and bidirectional LSTM with 1D convolution layers provide accurate personalized information about the daily routines of individuals. Significance: Capturing daily behavior patterns enables more accurate future BGC predictions in AID systems and improves BGC regulation.

## 1. Introduction

Time series data are widely used in many fields and various data-driven modeling techniques are developed to represent the dynamic characteristics of systems and forecast the future behavior. The growing research in artificial intelligence has provided powerful machine learning (ML) techniques to contribute to data-driven model development. Real-world data provides several challenges to modeling and forecasting, such as missing values and outliers. Such imperfections in data can reduce the accuracy of ML and the models developed. This necessitates data preprocessing for imputation of missing values, down-and up-sampling, and data reconciliation. Data preprocessing is a laborious and time-consuming effort since big data are usually stacked on a large scale [1]. When models are used for forecasting, the accuracy of forecasts improve if the effects of future possible disturbances based on behavior patterns extracted from historical data are incorporated in the forecasts. This paper focuses on these two problems and investigates the benefits of preprocessing the real-world data and the performance of different recurrent neural network (RNN) models for detecting various events that affect blood glucose concentration (BGC) in people with type 1 diabetes (T1D). The behavior patterns detected are used for more accurate predictions of future BGC variations, which can be used for warnings and for increasing the effectiveness of automated insulin delivery (AID) systems.

Time series data captured in daily living of people with chronic conditions have many of these challenges to modeling, detection, and forecasting. Focusing on people with T1D, the medical objective is to forecast the BGC of a person with T1D and prevent the excursion of BGC outside a “desired range” (70–180 mg/dL) to reduce the probability of hypo- and hyperglycemia events. In recent years, the number of people with diabetes has grown rapidly around the world, reaching pandemic levels [2,3]. Advances in continuous glucose monitoring (CGM) systems, insulin pump and insulin pen technologies, and in novel insulin formulations enabled many powerful treatment options [4–9]. The current treatment options available to people with T1D range from manual insulin injections to AID. Manual injection (insulin bolus) doses are computed based on the person’s characteristics and the properties of the meal consumed. Current AID systems necessitate manual entry of meal information to give insulin boluses for mitigating the effects of meal on the BGC. Manual adjustment of basal insulin dose, increasing the BGC target level and/or consumption of snacks are the options to mitigate the effects of physical activity. Some people may forget to make these manual entries and a system that can nudge them for providing appropriate information can reduce the extreme excursions in BGC. Commercially available AID systems are hybrid closed-loop systems and they require these manual entries by the user. AID systems, also called artificial pancreas (AP), consist of a CGM, an insulin pump, and a closed-loop control algorithm that manipulates the insulin infusion rate delivered by the pump based on the recent CGM values reported [10–23]. More advanced AID systems that use a multivariable approach [10,24–26] use additional inputs from a wearable devices (such as wristbands) to automatically detect the occurrence of physical activity and incorporate this information to the automated control algorithms for a fully-automated AID system [27]. Most AID systems use model predictive control techniques that predict future BGC values in making their insulin dosing decisions. Knowing the habits of the individual AID user improves the control decisions since the prediction accuracy of the future BGC trajectories can explicitly incorporate the future potential disturbances to the BGC, such as meals and physical activities, that will occur with high likelihood during the future BGC prediction window [24,26]. Consequently, the detection of meal and physical activity events from historical free-living data of a person with T1D will provide useful information for decision-making by both the individual and by the AID system.

CGM systems report subcutaneous glucose concentration to infer BGC with a sampling rate of 5 minutes. Self-reported meal and physical activity data are often based on diary entries. Physical activity data can also be captured by wearable devices. The variables reported by wearable devices may have artifacts, noise, missing values, and outliers. The data used in this work includes only CGM values, insulin dosing information, and diary entries of meals and physical activities.

**Figure 1.**
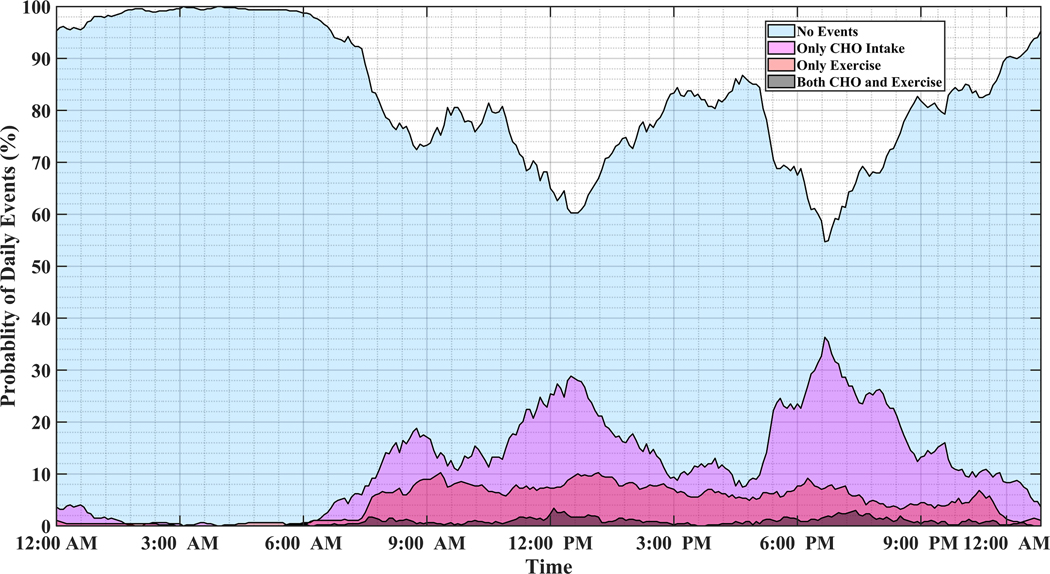
The probabilities of meal and physical activity events during one day obtained by analyzing 15 months of the pump-CGM sensor, meal, and physical activity data collected from a randomly selected person with T1D.

Analyzing long-term data of people with T1D indicates that individuals tend to repeat daily habitual behaviors. Figure 1 illustrates the probability of physical activity and meal (indicated as carbohydrate intake) events, either simultaneously or disjointly, for 15 months of CGM, meal, insulin pump, and physical activity self-recorded data of individuals with T1D. Major factors affecting BGC variations usually occur at specific time windows and conditions, and some combinations of events are mutually exclusive. For example, insulin-bolusing and physical activity are less likely to occur simultaneously or during hypoglycemia episodes, since people do not exercise when their BGC is low. People may have different patterns of behavior during the work week versus weekends or holidays. Predicting the probabilities of exercise, meal consumption, and their concurrent occurrence based on historical data using ML can provide important information on the behavior patterns for making medical therapy decisions in diabetes.

Motivated by the above considerations, this work develops a framework for predicting the probabilities of meal and physical activity events, including their independent and simultaneous occurrences. A framework is built to handle the inconsistencies and complexities of real-world data, including missing data, outlier removal, feature extraction, and data augmentation. Four different recurrent neural network (RNN) models are developed and evaluated for estimating the probability of events causing large variations in BGC. The advent of deep neural networks (NN) and their advances have had paved the way for processing and analyzing various types of information, namely: time-series, spatial, and time series-spatial data. Long short-term memory (LSTM) NN models are specific sub-categories of recurrent NNs introduced to reduce the computational burden of storing information over extended time intervals [28,29]. LSTMs take advantage of nonlinear dynamic modeling without knowing time-dependency information in the data. Moreover, their multi-step ahead prediction capability makes them an appropriate choice for detecting upcoming events and disturbances that can deteriorate the accuracy of model predictions.

The main contributions of this work are the development of NN models capable of estimating the occurrences of meals and physical activities without requiring additional bio-signals from wearable devices, and the integration of convolution layers with LSTM that enable the NN to accurately estimate the output from glucose-insulin input data. The proposed RNN models can be integrated with the control algorithm of an AID system to enhance its performance by readjusting the conservativeness and aggressiveness of the AID system.

The remainder of this paper is organized as follows: The next section provides a short description of the data collected from people with T1D. The preprocessing step, including outlier removal, data imputation, and feature extraction is presented in Section 3. Section 4 presents various RNN configurations used in this study. A case study with real-world data and a discussion of the results are presented in Sections 5 and 6, respectively. Finally, Section 6 provides the conclusions.

## 2. Free-living, Self-reported Dataset of People with T1D

A total of 300 self-collected T1D datasets were made available for research, and each dataset represents a unique individual. Among all the datasets, 50 T1D datasets include CGM sensor-insulin pump recordings and exercise information such as the time, type, and duration of physical activity recorded from either open/closed-loop insulin pump-sensor data. Meal information is reported as amount of carbohydrates (CHO) consumed in the meal as estimated by the subject. Over or underestimation of CHO in meals is common.

The subjects with T1D selected for this study used insulin pump-CGM sensor therapy for up to two years, and some of them have lived with diabetes for more than fifty years Tables 1 and 2 summarize the demographic information of the selected subjects and the definition of the variables collected, respectively. Separate RNN models are developed for each person in order to capture personalized patterns of meal consumption and physical activity.

## 3. Data Preprocessing

Using real-world data for developing models usually has numerous challenges: (i) the datasets can be noisy and incomplete; (ii) there may be duplicate CGM samples in some of the datasets; (iii) inconsistencies exist in the sampling rate of CGM and insulin values; (iv) gaps in the time and date can be found due to insulin pump or CGM sensor disconnection. Therefore, the datasets need to be preprocessed before using them for model development.

### 3.1. Sample Imputation

Estimating missing data is an important step before analyzing the data [30]. Missing data is substituted with reasonable estimates (imputation) [31]. In dealing with time-series data such as CGM, observations are sorted according to their chronological order. Therefore, the variable “Time”, described in Table 2, is converted to “Unix time-stamp” and samples are sorted in ascending order of “Unix time-stamp” and gaps without observations are filled with pump-sensor samples labeled as “missing values.”

Administered basal insulin is a piecewise constant variable and its amount is calculated by the AID system or by predefined insulin injection scenarios. Applying a simple forward or backward imputation for basal insulin with gaps in duration lasting a maximum of two hours gives reasonable reconstructed values for the missing observations. Gaps lasting more than two hours in missing recordings are imputed with basal insulin values recorded in the previous day at the same time, knowing that insulin injection scenarios usually follow a daily pattern [32].

Variable “Bolus” is a sparse variable (usually nonzero only at times of meals) and its missing samples are imputed with the median imputation approach, considering that bolus injection policy is infrequently altered. Similarly, missing recordings of variables “Nutrition.carbohydrate”, “Smbg”, “Duration”, “Activity.duration”, and “Distance.value” are imputed with the median strategy. A multivariate strategy, which uses CGM, total injected insulin, “Nutrition.carbohydrate”, the “Energy.value”, and “Activity.duration”, is employed to impute missing CGM values.

This choice of variables has to do with the dynamic relationship between CGM and the amount of carbohydrate intake, the duration and the intensity of physical activity, and total injected insulin. Estimates of missing CGM samples are obtained by performing probabilistic principal component analysis (PPCA) on the lagged matrices of the CGM data. PPCA is an extension of principal component analysis where the Gaussian conditional distribution of the latent variables is assumed [33]. This formulation of the PPCA facilitates tackling the problem of missing values in the data through the maximum likelihood estimation of the mean and variance of the original data. Before performing PPCA on the feature variables, the lagged array of each feature variable, $X_{k,j},k\in \{CGM,Ins,CHO,EV,AD\}$, at the j^*th*^ sampling index is constructed from the past two hours of observations as:

(1) $$X_{k,j}={\left[X_{k,j},X_{k,j-1}\ldots X_{k,j-24}\right]}_{1\times 25^{\prime }}\;k\in \{CGM,Ins,CHO,EV,AD\}\;X_{j}={\left[X_{1,j},\ldots ,X_{k,j}\ldots ,X_{M,j}\right]}^{T},\;X={\left[X_{1},\ldots ,X_{N}\right]}_{M\times N}$$

For an observed set of feature variables $X_{j}$, let $T_{j}={\left[T_{1,j},\ldots ,T_{q,j}\right]}^{T}$ be its q-dimensional (q≤M) Gaussian latent transform [34] such that

(2) $$X_{i,j}=W_{i}T_{j}+\mu_{i}+\epsilon_{i,j}$$

where $W_{i}=\left[W_{i,1},\ldots ,W_{i,q}\right]\in {\mathbb{R}}^{q}$ and $\underline{\mu }={\left[\mu_{1},\ldots ,\mu_{M}\right]}^{T}\in {\mathbb{R}}^{M}$ represent the *i^th^* row of the loading matrix $W\in {\mathbb{R}}^{M\times q}$ and mean value of the data. $\epsilon_{i,j}\in \mathbb{R}$ is also the measurement noise with the probability distribution

(3) $$p\left(\epsilon_{i,j}|\sigma^{2}\right)=N\left(\epsilon_{i,j}|0,\sigma^{2}\right).$$

Based on the Gaussian distribution assumption of $T_{j}$, and the Gaussian probability distribution of $\epsilon_{i,j}$, one can deduce that

(4) $$\{\begin{array}{l} p\left(T_{j}\right)=N\left(T_{j}|0,I_{q}\right)\\ p\left(X_{i,j}|\mu_{i},W_{i},\sigma^{2}\right)=N\left(X_{i,j}|\mu_{i},W_{i}W_{i}^{T}+\sigma^{2}\right)\\ p\left(X_{i,j}|T_{j},\mu_{i},W_{i},\sigma^{2}\right)=N\left(X_{i,j}|W_{i}T_{j}+\mu_{i},\sigma^{2}\right) \end{array}.$$

The joint probability distribution $p\left(X_{i,j},T_{j},\mu_{i},W_{i},\sigma^{2}\right)$ can be derived from (4) and Bayes’ joint probability rule as

(5) $$p\left(X_{i,j},T_{j},\mu_{i},W_{i},\sigma^{2}\right)=\frac{1}{{\left(2\pi \sigma^{2}\right)}^{\frac{M}{2}}}\exp\frac{{\left(X_{i,j}-W_{i}T_{j}-\mu_{i}\right)}^{2}}{-2\sigma^{2}}\frac{1}{{\left(2\pi \right)}^{\frac{q}{2}}}\exp\frac{-T_{j}^{T}T_{j}}{2}$$

Define the set $\eta =\left\{(i,j)|1\leq i\leq M,1\leq j\leq N,X_{i,j}\neq NaN\right\}$. The log-likelihood of the joint multivariate Gaussian probability distribution of (5) is calculated over all available observations as

(6) $$ln\left(p\left(X_{i,j},T_{j}|\mu_{i},W_{i},\sigma^{2}\right)\right)=\sum_{i,j\in \eta }\sum \left[\ln\left(p\left(X_{i,j}|T_{j},\mu_{i},W_{i},\sigma^{2}\right)\right)+\ln p\left(T_{j}\right)\right]\;\;=\sum_{i,j\in \eta }\sum \frac{-M}{2}\ln\left(2\pi \sigma^{2}\right)-\frac{q}{2}\ln(2\pi )-\frac{{\left(X_{i,j}-W_{i}T_{j}-\mu_{i}\right)}^{2}}{2\sigma^{2}}-\frac{T_{j}^{T}T_{j}}{2}$$

where the log-likelihood (6) is defined for all available observations $X_{i,j}$, i,j∈η. By applying the expectation operation with respect to the posterior probability distribution over all latent variables $T_{j}$, $j\in \eta_{i}$, where $\eta_{i}=\left\{j|1\leq j\leq N,X_{i,j}\neq NaN\right\}$ (6) becomes

(7) $$E\{L\}=-\sum_{i,j\in \eta }\sum \frac{M}{2}ln\left(\sigma^{2}\right)+\frac{1}{2}E\left\{T_{j}^{T}T_{j}\right\}+\frac{1}{2\sigma^{2}}{\left(X_{i,j}-\mu_{i}\right)}^{2}\;\;-\frac{1}{\sigma^{2}}E\left\{T_{j}^{T}\right\}W_{i}^{T}\left(X_{i,j}-\mu_{i}\right)+\frac{1}{2\sigma^{2}}E\left\{T_{j}^{T}T_{j}\right\}W_{i}W_{i}^{T}$$

Maximizing (7) is feasible by setting all partial derivatives $\frac{\partial E\{L\}}{\partial \sigma^{2}}$, $\frac{\partial E\{L\}}{\partial \mu_{i}^{2}}$, and $\frac{\partial E\{L\}}{\partial W_{i}^{2}}$, i=1,…,M, j=1,…,N to zero [34].


(8) $$Cvar_{T_{j}}=\frac{\sigma^{2}}{\left(\sigma^{2}I_{q}+\sum_{i\in \eta_{j}}W_{i}W_{i}^{T}\right)}\;\mu_{T_{j}}=\frac{Cvar_{T_{j}}}{\sigma^{2}}\sum_{i\in \eta_{j}}W_{i}^{T}\left(X_{i,j}-\mu_{i}\right)\;\mu_{i}=\frac{1}{\left|\eta_{i}\right|}\sum_{j\in \eta_{i}}\left[X_{i,j}-W_{i}\mu_{T_{j}}\right]\;W_{i}=\frac{1}{\sum_{j\in \eta_{i}}\left[\mu_{T_{j}}\mu_{T_{j}}^{T}+Cvar_{T_{j}}\right]}\sum_{j\in \eta_{i}}\mu_{T_{j}}\left(X_{i,j}-\mu_{i}\right)\;\sigma^{2}=\frac{1}{\left|\eta \right|}\sum_{i,j\in \eta }\left[{\left(X_{i,j}-W_{i}\mu_{T_{j}}-\mu_{i}\right)}^{2}+W_{i}Cvar_{T_{j}}W_{i}^{T}\right]$$

Parameters $\mu_{i}$, $\sigma^{2}$, and $W_{i}$ in (8) are updated recursively until they converge to their final values. The final estimation of missing CGM samples is obtained by performing diagonal averaging of the reconstructed lagged matrix $\hat{X}\in {\mathbb{R}}^{M\times N}$ over rows/columns filled with CGM values. Long gaps in CGM recordings might exist in the data, and imputing their values causes problems in accuracy and reliability. Therefore, CGM gaps no more than twenty-five consecutive missing samples (about two hours) are imputed by PPCA.

**Algorithm 1:** Outlier rejection from CGM readings:

1:	**procedure** Outlierrejectoon(*CGM, Smbg, CHO*, *AD*, *Ins_Bolus_*)	
2:	**for** *i* = 1 : *N* **do**	▹ Removing samples outside of the calibration range
3:	**if** $CGM_{k}>400\;\text{mg}/\text{dL}$ or $CGM_{k}<0\;\text{mg}/\text{dL}$ **then**	
4:	$CGM_{k}\leftarrow NaN$	
5:	**end if**	
6:	**end for**	
7:	**for** *i* = 2 : *N* **do**	
8:	$\text{Δ}CGM_{k}\leftarrow CGM_{k}-CGM_{k-1}$	
9:	**if** $\text{Δ}CGM_{k}>30\;\text{mg}/\text{dL}$ & all $\left(\left\{{\text{CHO}}_{k},\ldots ,CHO_{k-9}\right\}==0\right)$ **then**	
10:	$CGM_{k}\leftarrow \text{NaN}$	
11:	**end if**	
12:	**if** $\text{Δ}CGM_{k}<30\;\text{mg}/\text{dL}$ & all $\left(\left\{{\text{Ins}}_{Bolus,k},\ldots ,\ldots ,{\text{Ins}}_{Bolus,k-6}\right\}==0\right)$ **then**	
13:	$CGM_{k}\leftarrow \text{NaN}$	
14:	**end if**	
15:	**if** $\text{Δ}CGM_{k}<30\;\text{mg}/\text{dL}$ & all $\left(\left\{AD_{k},\ldots ,AD_{k-6}\right\}==0\right)$ **then**	
16:	$CGM_{k}\leftarrow \text{NaN}$	
17:	**end if**	
18:	**if** $Smbg_{k}\neq \text{NaN}$ & $CGM_{k}\neq \text{NaN}$ & $\text{abs}\left(Smbg_{k}-CGM_{k}\right)>18\;\text{mg}/dL$ **then**	
19:	${\text{CGM}}_{k}\leftarrow \text{NaN}$	
20:	**end if**	
21:	**end for**	
22:	**return** CGM	
23:	**end procedure**	

### 3.2. Outlier Removal

Signal reconciliation and outlier removal are necessary to avoid misleading interpretation of data and biased results, and to improve the quality of CGM observations. As a simple outlier removal approach for a variable with Gaussian distribution, observations outside ±2.72 standard deviations from the mean, known as Inner Tukey Fences, can be labeled outliers and extreme values [35]. The probability distribution of the CGM data shows a skewed distribution compared to the Gaussian probability distribution. Thus, labeling samples as outliers only based on their probability of occurrence is not the proper way of removing extreme values from the CGM data since it can cause loss of useful CGM information, specifically during hypoglycemia (CGM<70mg/dL) and hyperglycemia (CGM>180mg/dL) events. As another alternative, extreme values, and spikes in the CGM data can be labeled from the prior knowledge and by utilizing other feature variables, namely: “Smbg,” “Nutrition.carbohydrate,” “Bolus,” and “Activity.duration.” Algorithm 1 is proposed to remove outliers from CGM values. Usually, BGC is slightly different from the recordings of CGM signal because of delay between BGC and subcutaneous glucose concentration measured by the CGM device and sensor noise. The noisy signal can deteriorate the performance of data-driven models. Therefore, Algorithm 2, which is based on eigendecomposition of the Hankel matrix of CGM values, is used to reduce the noise in the CGM recordings.

### 3.3. Feature Extraction

Converting raw data into informative feature variables or extracting new features is an essential step of data preprocessing. In this study, four groups of feature variables, including frequency domain, statistical domain, nonlinear domain, and model-based features are calculated and added to each dataset to enhance the prediction power of models. The summarized description of each group of features and the number of past samples required for their calculation are listed in Table 3.

Qualitative trend analysis of variables can extract different patterns caused by external factors within a specified time [36,37]. A pairwise multiplication of the sign and magnitude of the first and second derivatives of CGM values indicates carbohydrate intake [38,39], exogenous insulin injection, and physical activity. Therefore, the first and second derivatives of CGM values, calculated by the fourth-order backward difference method are added as feature variables. The sign and magnitude product of the first and second derivatives of CGM, their covariance, Pearson correlation coefficient, and Gaussian kernel similarity are extracted. Statistical feature variables, e.g., mean, standard deviation, variance, skewness, etc., are obtained from the specified time window of CGM values. Similar to the first and second derivatives of CGM values, a set of feature variables, including covariance and correlation coefficients, from pairs of CGM values and derivatives are extracted and augmented to the data.

Because of the daily repetition in the trends of CGM and glycemic events, and the longer time window of CGM values, samples collected during the last twenty-four hours are used for frequency-domain feature extraction. Therefore, magnitudes and frequencies of the top three dominant peaks in the power spectrum of CGM values, conveying past long-term variation of the BGC, are included in the set of feature maps.

**Algorithm 2:** Smoothing CGM recordings:

1:	**procedure** cgmdenoising(*CGM*)	▹ Smoothing CGM recordings
2:	$Q_{i}=\left[CGM_{d},\ldots ,CGM_{d+q_{i}-1}\right]$	▹ $Q_{i}\in {\mathbb{R}}^{q_{i}}\;\text{is}\;i^{th}$ consecutive CGM recordings
3:	$q_{i}\leftarrow \left|Q_{i}\right|$, $p_{i}\leftarrow \text{floor}\left(\frac{q_{i}}{2}\right)$, $w_{i}\leftarrow q_{i}-p_{i}+1$	
4:	$\left[U_{i},S_{i},V_{i}\right]=SVD\left(A_{i}\right)$	▹ $A_{i}\in {\mathbb{R}}^{w_{i}\times p_{i}}$ is the Hankel matrix made of *Q_i_*
5:	${\hat{S}}_{i}\leftarrow \text{zeros}(p_{i},p_{i})$	
6:	$\eta \leftarrow \frac{cumsum\left(\left[s_{1},\ldots ,s_{p_{i}}\right]\right)}{sum\left(\left[s_{1},\ldots ,s_{p_{i}}\right]\right)}$	▹ $s_{j}>0$ are eigenvalues of $S_{i}$ in descending order
7:	**for** *j*=1: $p_{i}$ **do**	
8:	**if** $\eta_{j}>0.95$ **then**	
9:	${\hat{S}}_{i}(j,j)\leftarrow 0$	
10:	**else**	
11:	${\hat{S}}_{i}(j,j)\leftarrow S_{i}(j,j)$	
12:	**end if**	
13:	**end for**	
14:	${\hat{A}}_{i}=U_{i}{\hat{S}}_{i}V_{i}^{T}$	
15:	${\hat{Q}}_{i}\leftarrow \text{Diagonalaveraging}\left({\hat{A}}_{i}\right)$	▹ ${\hat{Q}}_{i}=\left[C\hat{G}M_{d},\ldots ,C\hat{G}M_{d+q_{i}-1}\right]$
16:	**return** $C\hat{G}M$	
17:	**end procedure**	

Plasma insulin concentration (PIC) is another feature variable that informs about the carbohydrate intake information and exogenous insulin administration. PIC accounts for the accumulation of subcutaneously injected insulin within the bloodstream, which is gradually consumed by the body to enable the absorption of carbohydrates released from the gastrointestinal track to various cells and tissues. Usually, dynamic physiological models are used to describe and model the glucose and insulin concentration dynamics in diabetes. The main idea of estimating PIC from physiological models stems from predicting the intermediate state variables of physiological models by designing a state observer and utilizing total infused insulin and carbohydrate intake as model inputs, and CGM values as the output of the model [40–42]. In this work, the estimation of PIC and glucose appearance rate are obtained from a physiological model known as Hovorka’s model [43]. Equation (9) presents this nonlinear physiological (compartment) model:

(9) $$\begin{array}{l} \frac{dS_{1}(t)}{dt}=Ins(t)-\frac{S_{1}(t)}{t_{\max,I}}\\ \frac{dS_{2}(t)}{dt}=\frac{S_{1}(t)}{t_{\max,I}}-\frac{S_{2}(t)}{t_{\max,I}}\\ \frac{dI(t)}{dt}=\frac{S_{2}(t)}{t_{\max,I}V_{I}}-K_{e}I(t)\\ \frac{dx_{1}(t)}{dt}=k_{b,1}I(t)-k_{a,1}x_{1}(t)\\ \frac{dx_{2}(t)}{dt}=k_{b,2}I(t)-k_{a,2}x_{2}(t)\\ \frac{dx_{3}(t)}{dt}=k_{b,3}I(t)-k_{a,3}x_{3}(t)\\ \frac{dQ_{1}(t)}{dt}=U_{g}(t)-F_{0,1}^{c}(t)-F_{R}(t)-x_{1}(t)Q_{1}(t)+k_{12}Q_{2}(t)+EGP_{0}\left(1-x_{3}(t)\right)\\ \frac{dQ_{2}(t)}{dt}=x_{1}(t)Q_{1}(t)-\left(k_{12}+x_{2}(t)\right)Q_{2}(t)\\ \frac{dG_{sub}(t)}{dt}=\frac{1}{\tau }\left(\frac{Q_{1}(t)}{V_{g}}-G_{sub}(t)\right) \end{array}$$

Model (9) is comprised of four sub-models, describing the action of insulin on glucose dynamics, the insulin absorption dynamics, plasma-interstitial tissue glucose concentration dynamics, and the blood glucose dynamics.The state variables of (9), the nominal values of the parameters, and their units are listed in Table 4 [43]

Body weight has a significant effect on the variations of the PIC and other state variables as it is used for determining the amount of exogenous insulin to be infused. Although estimating body weight as an augmented state variable of the insulin-CGM model is an effective strategy to cope with the problem of unavailable demographic information, estimating body weight from total amount of daily administered insulin is a more reliable approach. As reported in various studies, total daily injected insulin can have a range 0.4–1.0 *units. kg*^−1^.*day*^−1^ [44–46]. A fair estimation of body weight can be obtained by calculating the most common amount of injected basal/bolus insulin for each subject and using a conversion factor of 0.5 *units.kg*^−1^.*day*^−1^ as a rule of thumb to estimate the body weight.

The insulin-glucose dynamics (9) in discrete-time format are given by

(10) $$X_{k+1}^{\prime }=f^{\prime }\left(X_{k}^{\prime },U_{k}\right)+G_{k}\omega_{k},\;\omega_{k}\approx N(0,Q)\;Y_{k}^{\prime }=h^{\prime }\left(X_{k}^{\prime }\right)+v_{k},\;v_{k}\approx N(0,R)$$

where $X_{k}^{\prime }=\left[S_{1,k},S_{2,k},I_{k},x_{1,k},x_{2,k}x_{3,k},Q_{1,k},Q_{2,k},G_{sub,k},t_{\max,I,k},k_{e,k},U_{G,k}\right]\in R^{n_{x}}$ denotes the extended state variables and $U_{k}$ is the total injected exogenous insulin. Symbols $\omega_{k}$ and $\nu_{k}$ denote zero-mean Gaussian random process and measurement noises (respectively), representing any other uncertainty and model mismatch that are not taken into account.

Further, $Q\in R^{n_{x}\times n_{x}}$ and R∈R represent the positive definite system uncertainty and measurement noise covariance matrices, respectively.

Tracking the dynamics of internal state variables of the model (10) is feasible by using a class of Sequential Monte Carlo algorithms known as particle filters. A generic form of the particle filter algorithm proposed by [47] with efficient adaptive Metropolis-Hastings resampling strategy developed in [49] is employed to predict the trajectory of the PIC and other state variables. In order to avoid any misleading state estimations, each state variable is subjected to a constraint to maintain all estimations within meaningful intervals [41].

### 3.4. Feature Selection and Dimensionality Reduction

Reducing the number of redundant feature variables lowers the computational burden of their extraction and hinders over-parameterized modeling. In this work, a two-step feature selection procedure is used to obtain the optimal subset of feature variables that boost the efficiency of the classifier the most. In the first step, the deviance statistic test is performed to filter out features with low significance (P-value>0.05). In the second step, the training split of all datasets was used in the wrapper feature selection strategy to maximize the accuracy of the classifier in estimating the glycemic events. Sequential floating forward selection (SFFS) approach [50] was applied on a random forest estimator with thirty decision tree classifiers with a maximum depth of six layers to sort out features with the most predictive power in descending order. Consequently, the top twenty feature variables with the highest contribution to the classification accuracy enhancement are used for model development.

## 4. Detection and Classification Methods

Detecting the occurrence of events causing large glycemic variations requires solving a supervised classification problem. Hence, all samples required labeling using the information provided in the datasets, specifically using variables “Activity.duration” and “Nutrition.carbohydrate.” In order to determine index sets of each class, let *N* to be total number of samples and $T(k)=ceil\left(AD(k)/\left(3\times 10^{5}\right)\right)$ be the sample duration of physical activity at each sampling time k. Define sets of sample indexes as:

(11) $$\{\begin{array}{ll} \text{Label}{\text{.Index}}_{\left\{1,1\right\}}:= & \begin{array}{l} \{i|k\leq i\leq k+T(k)-1,\;k=1,\ldots ,N,\;T(k)\neq 0,\\ \text{Nutrition.carbohydrate}(j)\neq 0,\;j=k+1,\ldots ,k+T(k)\} \end{array}\\ \text{Label}{\text{.Index}}_{\left\{0,1\right\}}:= & \begin{array}{l} \{i|k\leq i\leq k+T(k)-1,\;k=1,\ldots ,N,\;T(k)\neq 0,\\ \text{Nutrition.carbohydrate}(j)=0,\;j=k+1,\ldots ,k+T(k)\} \end{array}\\ \text{Label}{\text{.Index}}_{\left\{1,0\right\}}:= & \left\{k|1\leq k\leq N,\;T(k)=0,\;\text{Nutrition.carbohydrate}(k)\neq 0\right\}\\ {\text{Label.Index}}_{\left\{0,0\right\}}:= & \left\{k|1\leq k\leq N,\;T(k)=0,\;\text{Nutrition.carbohydrate}(k)=0\right\} \end{array}$$

The label indexes defined by (11) corresponds to classes “Meal and Exercise,” “no Meal but Exercise,” “no Exercise but Meal,” “neither Meal nor Exercise,” respectively.

Four different configurations of the RNN models are studied to assess the accuracy 249 and performance of each in estimating the joint probability of the carbohydrate intake and physical activity. All four models use 24 past samples of the selected feature variables and

**Algorithm 3:** The generic particle filter algorithm [47,48]

1:	**procedure** ParticleFilter$\left(x_{k-1}=\left[x_{k-1}^{1},\ldots ,x_{k-1}^{N_{s}}\right],w_{k-1}=\left[w_{k-1}^{1},\ldots ,w_{k-1}^{N_{s}}\right],z_{k}\right)$	
2:	**if** *k* == 0 **then**	▹ Initialization step
3:	**for** *i* = 1 : $N_{s}$ **do**	
4:	$w_{0}^{i}\leftarrow \frac{1}{N_{s}}$	
5:	$x_{0}^{i}∼N(\eta ,\text{Σ})$	
6:	**end for**	
7:	**return** $x_{0}$, $w_{0}$	
8:	**else**	
9:	**for** *i* = 1 : $N_{s}$ **do**	
10:	$x_{k}^{i}∼\pi \left(x_{k}|x_{k-1}^{i},z_{k}\right)$	▹ Propagate particles
11:	$w_{k}^{i}\propto w_{k-1}^{i}\frac{p\left(y_{k}|x_{k}^{i}\right)p\left(x_{k}^{i}|x_{k-1}^{i}\right)}{\pi \left(x_{k}^{i}|x_{k-1}^{i},y_{k}\right)}$	▹ Update importance sampling weights
12:	**end for**	
13:	**for** *i* = 1 : $N_{S}$ **do**	
14:	$w_{k}^{i}\leftarrow \frac{w_{k}^{i}}{\sum_{j=1}^{N}w_{k}^{j}}$	▹ Normalize importance sampling weights
15:	**end for**	
16:	${\hat{N}}_{eff}\leftarrow \frac{1}{\sum_{j=1}^{N}{\left(w_{k}^{j}\right)}^{2}}$	▹ Calculate the effective sampling size
17:	**if** ${\hat{N}}_{eff}<N_{s}$ **then**	▹ Check for degeneracy issue
18:	$\left[x_{k}^{*},w_{k}^{*}\right]=\text{Resample}\left(x_{k}=\left[x_{k}^{1},\ldots ,x_{k}^{N_{s}}\right],w_{k}=\left[w_{k}^{1},\ldots ,w_{k}^{N_{s}}\right]\right)$	▹ Resample particles[49]
19:	$x_{k}\leftarrow x_{k}^{*}$	
20:	$w_{k}\leftarrow w_{k}^{*}$	
21:	**end if**	
22:	**return** $x_{k}$, $w_{k}$	
23:	**end if**	
24:	**end procedure**	

event estimations are performed one sample backward. Estimating the co-occurrences of the external disturbances should be performed at least one step backward as the effect of disturbance variables needs to be seen first, and then, parameter adjustment and event prediction can be made.

Since the imputation of gaps with a high number of consecutive missing values adversely affect the prediction of meal-exercise classes, all remaining samples with missing values after the data imputation step are excluded from parameter optimization. Excluding missing values inside the input tensor can be done either by using a placeholder for missing samples and filtering samples through masking layer or manually removing incomplete samples.

The first NN model consists of a masking layer to filter out unimputed samples, followed by a LSTM layer, two dense layers, and a softmax layer to estimate the probability of each class. The LSTM and dense layers are undergo training with dropout and parameter regularization strategies to avoid the drastic growth of hyperparameters. Additionally, the recurrent information stream in the LSTM layer was randomly ignored in the calculation at each run. At each layer of the network, the magnitude of both weights and intercept coefficients was penalized by adding a Li regularizer term to the loss function. The Rectified Linear Unit (ReLu) activation function was chosen as a nonlinear component in all layers. The input variables of the regular LSTM network will have the shape of N×m×L, that denotes the size of samples, the size of lagged samples, and the number of feature variables, respectively.

The second model encompasses a series of two 1D convolution layers, each one followed by a max pool layer for downsampling feature maps. The output of the second max pool layer was flattened to achieve time-series extracted feature to feed to to the LSTM layer. A dense layer after LSTM was added to the model and the joint probability of events estimated by calculating the output of the softmax layer. Like the first RNN model, the ReLU activation function was employed in all layers to capture the nonlinearity in the data. *L*_1_ regularization method was applied to all hyperparameters of the model. Adding convolution layers with repeated operations to an RNN model paves the way for extracting features for sequence regression or classification problem. This approach has shown a breakthrough in visual time-series prediction from the sequence of images, or videos, for various problems such as activity recognition, textual description, and audio and word sequence prediction [52,53]. Time-distributed convolution layers scan and elicit features from each block of the sequence of the data [54]. Therefore, each sample is reshaped into m×n×L, with *n* = 1 blocks at each sample.

**Figure 2.**
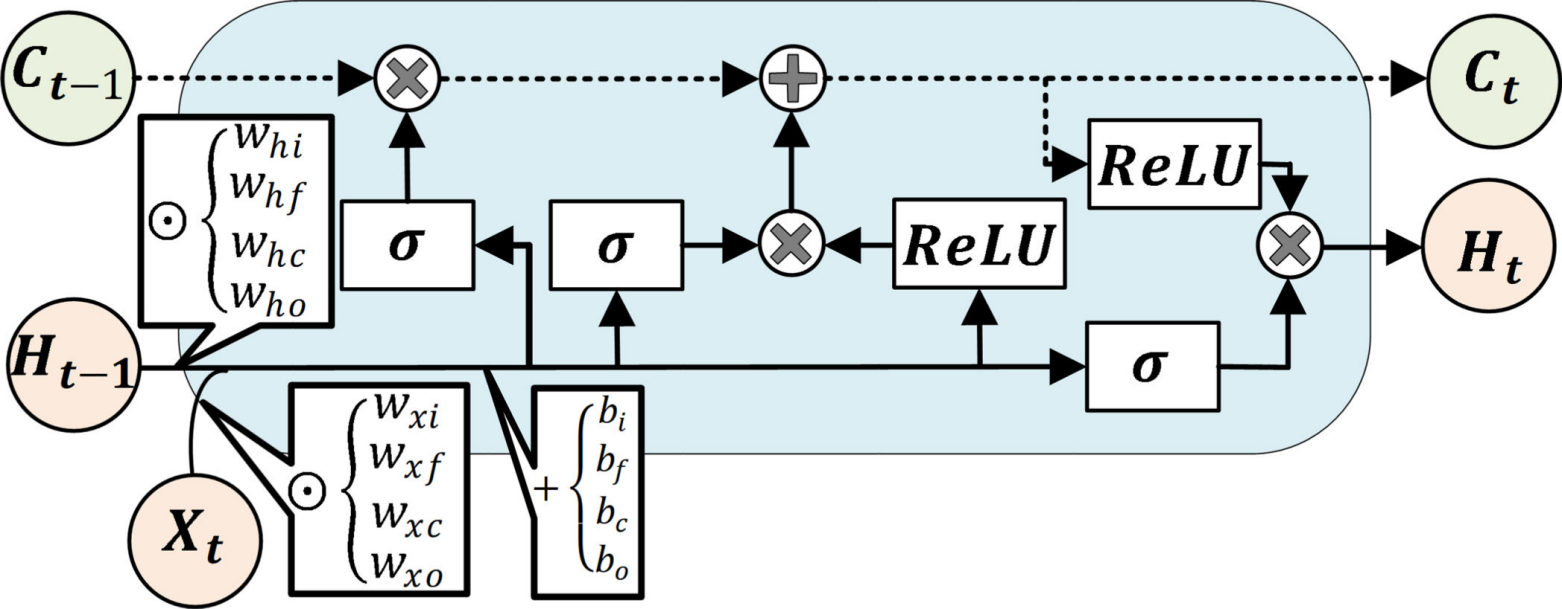

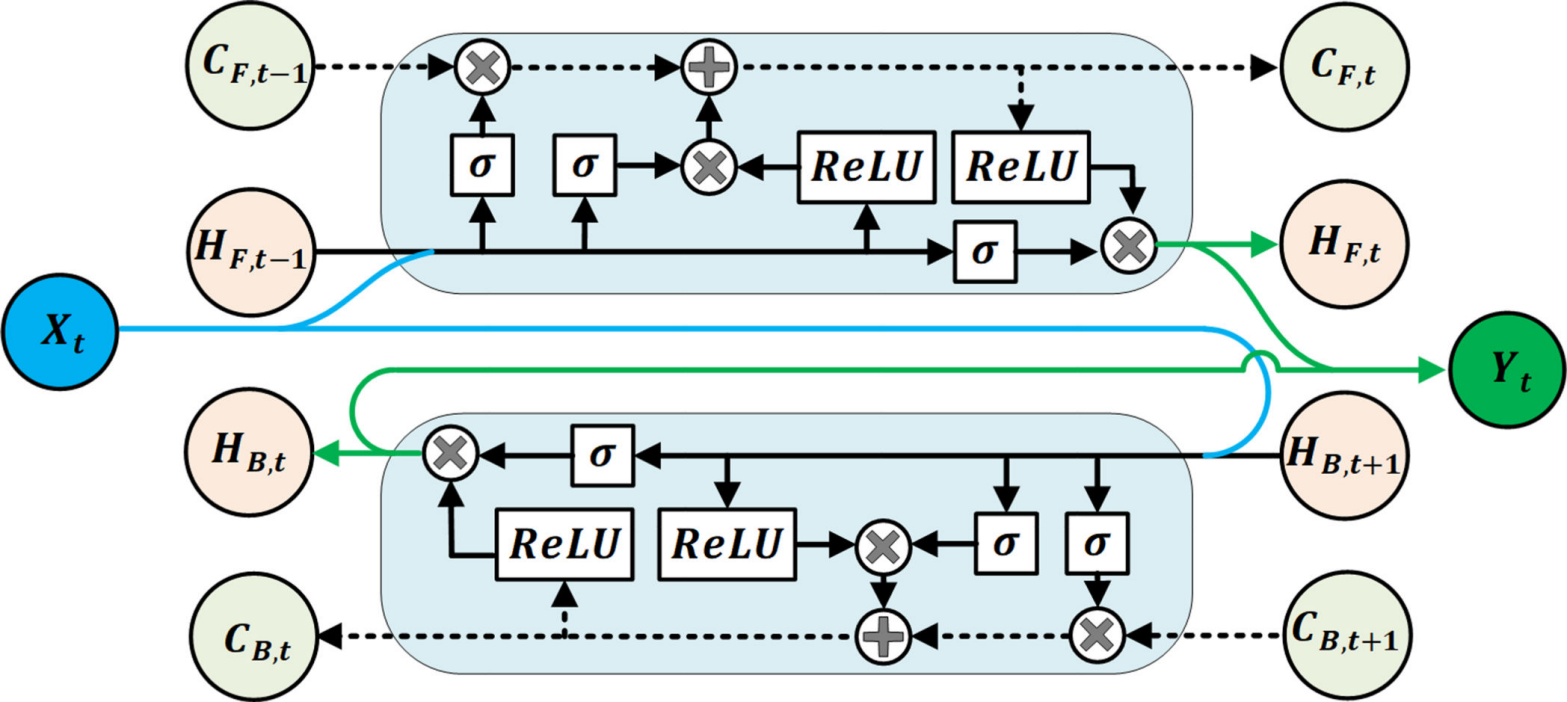

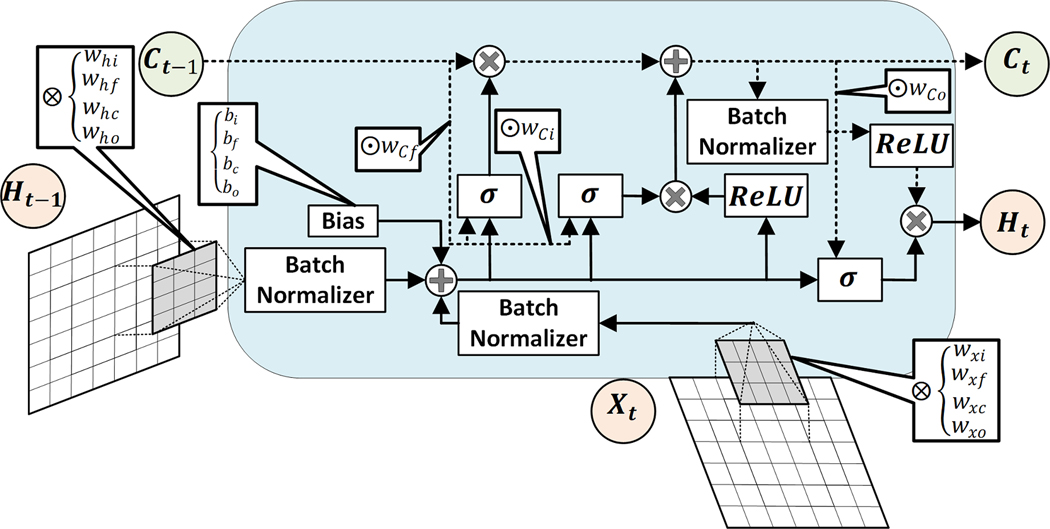
Structures of a regular LSTM unit (a), a Bi-LSTM unit (b), and schematic demonstration of a 2D ConvLSTM cell (c) [51].

The third classifier has a 2D convolutional LSTM (ConvLSTM) layer, one dropout layer, two dense, and a softmax layer for probability estimation of each class from the sequences of data. 2-D ConvLSTM structure was designed to capture both temporal and spatial correlation in the data, moving pictures in particular, by employing convolution operation in both input-to-state, and state-to-state transitions [51]. In comparison to a regular LSTM cell, ConvLSTMs perform convolution operation by internal multiplication of inputs and hidden states into kernel filter matrices (Figure 2 c). Similar to previously discussed models, L_1_ regularization constraint and ReLU activation function are considered in constructing the ConvLSTM model. 2D ConvLSTM import sample of spatiotemporal data in the format of m×s×n×L, where *s* = 1 and *n* = 1 stand for the size of the rows and columns of each tensor and *L* = 20 is the number of channels/features on the data [55].

Finally, the last model is comprised of two 1D convolution layers, two max pooling layers, a flatten layer, a bidirectional LSTM (Bi-LSTM) layer, a dense layer, and soft max layer to predict classes. Bi-LSTM units capture the dependency in the sequence of the data in two directions. Hence, as a comparison to a regular LSTM memory unit, Bi-LSTM requires to reversely duplicate the same LSTM unit and employ a merging strategy to calculate the output of the cell [56]. The use of this approach was primarily observed in speech recognition tasks, where instead of real-time interpretation, the whole sequence of the data was analyzed and its superior performance over the regular LSTM was justified [57]. The joint estimation of glycemic events is made one step backward. Therefore, the whole sequence of features are recorded first, and use of an RNN model with Bi-LSTM units for the detection of unannounced disturbances is quite justifiable. The tensor of input data is similar to LSTM with 1D convolutional layers. Figure 2 is the schematic diagram of a regular LSTM, a Bi-LSTM, and a ConvLSTM unit.

**Figure 3.**
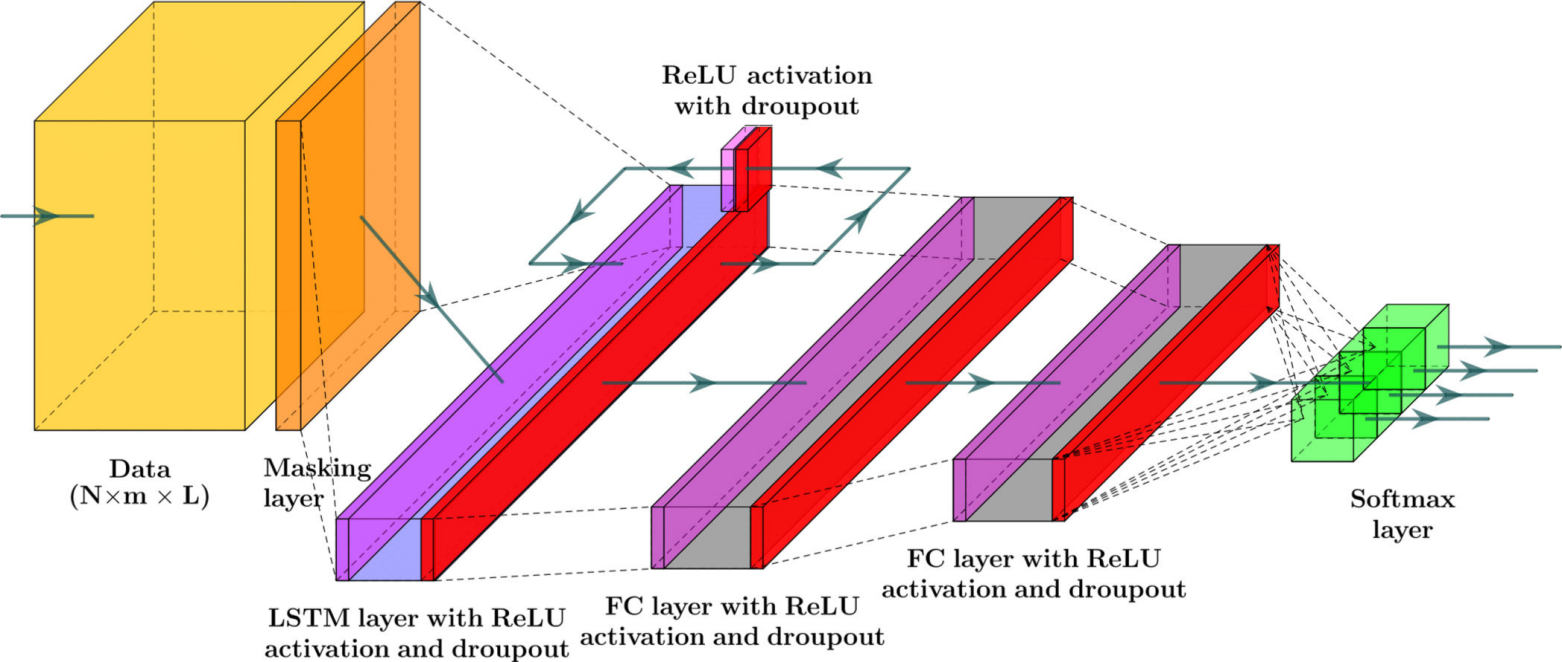

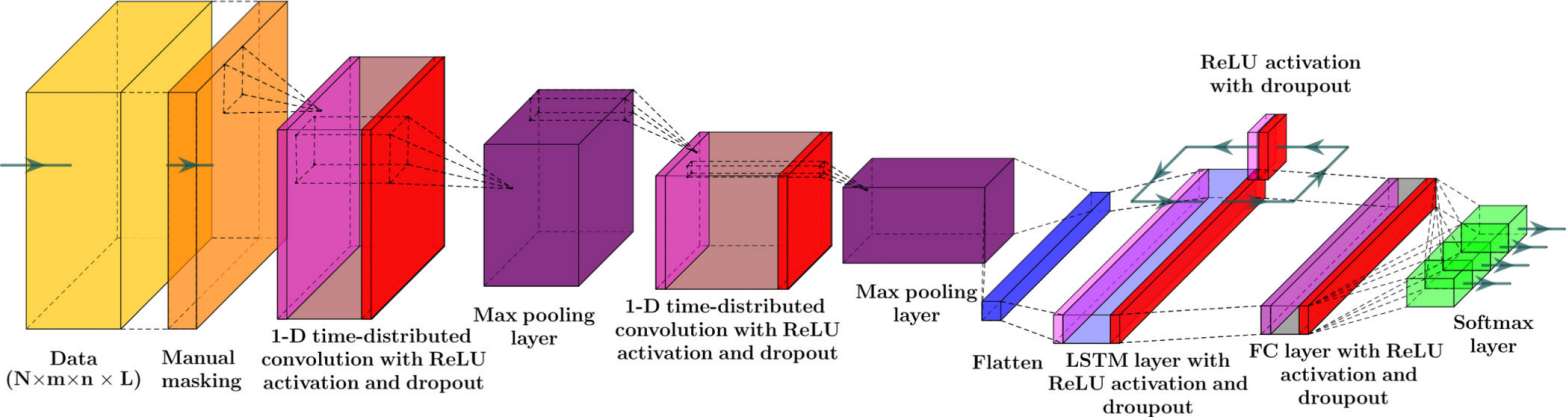

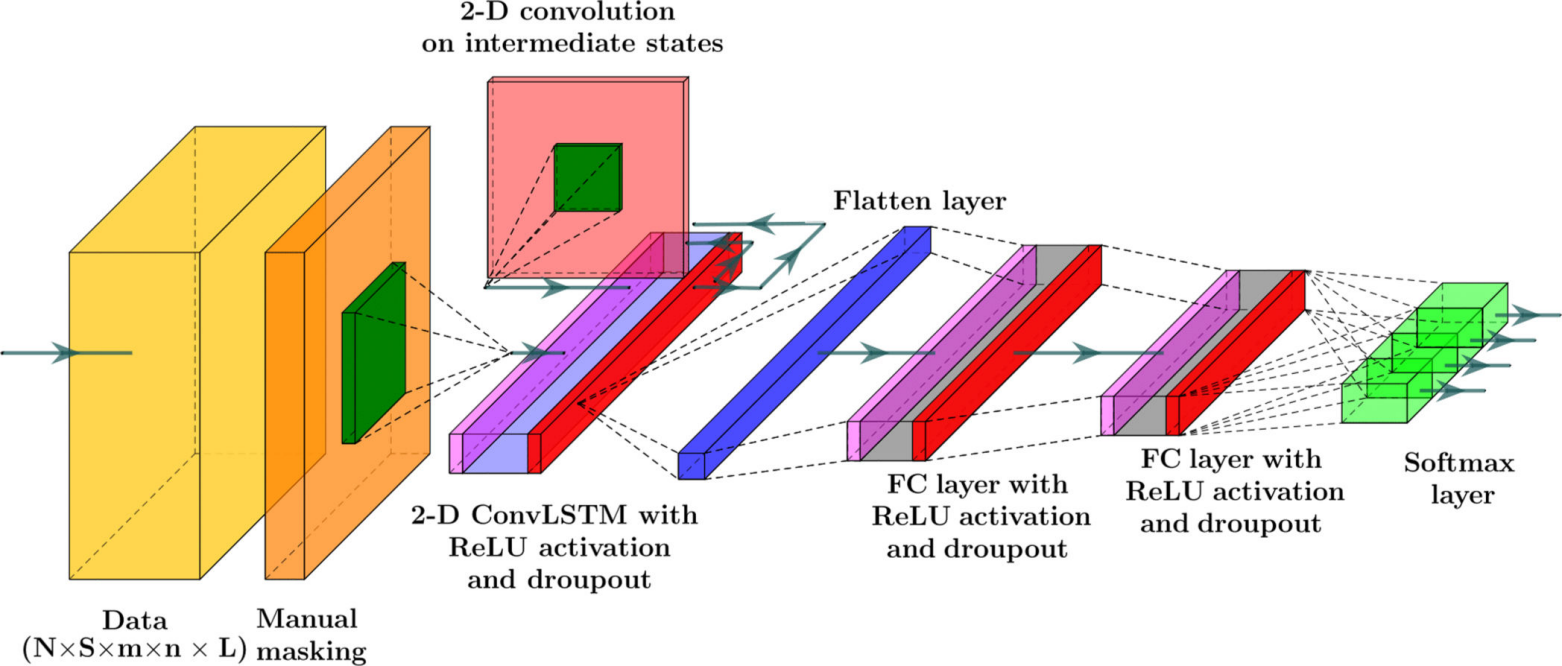

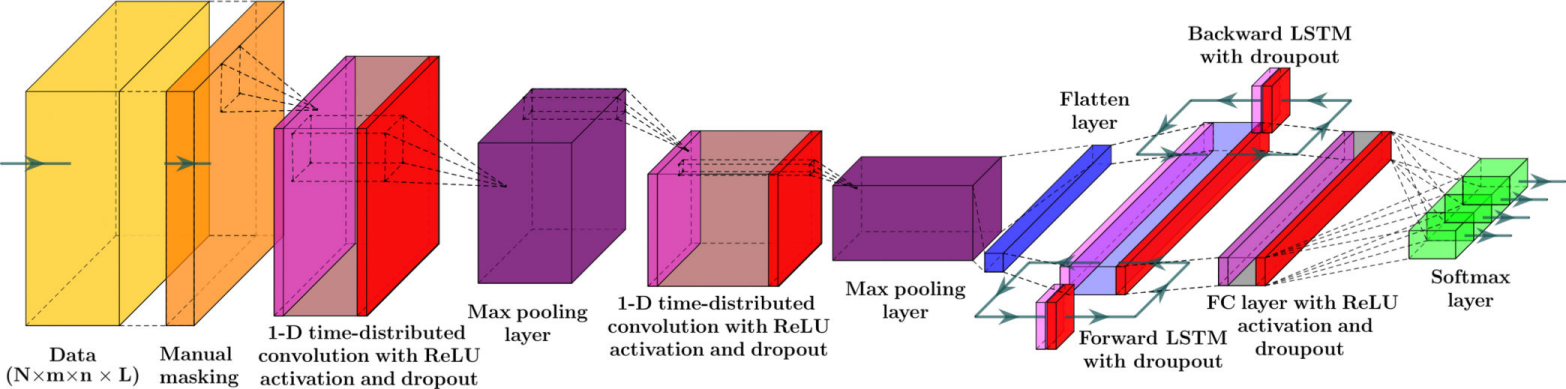
Systematic structures of the different RNN models included in the study: (a) LSTM NN model, (b) LSTM with 1D convolutional layers, (c) 2D ConvLSTM NN model, and (d) Bi-LSTM with 1D convolutional layers. Color dictionary: Yellow: tensor of data, Orange: Masking to exclude missing samples, Magenta: Relu activation, Light blue: LSTM layer, Red: dropout, Grey: dense layer, Green: softmax activation, Blue: flatten layer, Purple: max pool layer, Dark green: kernel filter, Light red: the matrix of intermediate states.

Figure 3 depicts the structure of the four RNN models to estimate the probability of meal consumption, physical activity, and their concurrent occurrence. The main difference between models (a) and (b) in Figure 3 is convolution and max-pooling layers added before the LSTM layer to extract features map from time series data. Although adding convolutional blocks to an RNN model increases the number of learnable parameters, including weights, biases, and kernel filters, calculating temporal feature maps from input data enhances better discriminates the target classes.

## 5. Case Study

Eleven datasets containing CGM sensor-insulin pump, physical activity, and carbohydrate intake information are selected randomly from subject records for a case study. Data imputation and reconciliation, RNN training, and evaluation of results are conducted individually for each subject. Hence, the RNN models are personalized, using only that person’s data. All datasets are preprocessed by the procedure elaborated in the data preprocessing section and feature variables are rescaled to have zero-mean and unit variance. Stratified six-fold cross-validation is applied to 87.5% of samples of each dataset to reduce the variance of predictions. Weight values proportional to the inversion of class sizes are assigned to the corresponding samples to avoid biased predictions caused by imbalanced samples in each class. In order to better assess the performance of each model and to avoid the effects of randomization in the initialization step of back propagation algorithm, each model is trained five times with different random seeds. Hyperparameters of all models are obtained through adaptive moment estimation (Adam) optimization algorithm and 2% of the training sample size was chosen as the size of the training batches. In model training with different random seeds, the number of adjustable parameters, including weights, biases, the size and number of filter kernels, and the learning rate remain constant.

One difficulty associated with convolution layers in models (b) and (d) is the optimization of the hyperparameters of the convolutional layers. Usually, RNN models with convolution layers require a relatively high computation time. As a solution, learning rates with small values are preferred for networks with convolutional layers since they lead to a more optimal solution compared to large learning weights which may result in non-optimality and instability.

Data preprocessing part of the work was conducted in Matlab 2019a environment and Keras/Keras-gpu 2.3.1 are used to construct and train all RNN models. Keras is a high-class API library with Tensorflow as the backend, all are available on Python environment. We have used two computational resources for data preparations and model training. Table 4 provides the details of hardware resources.

## 6. Discussion of Results

Each classifier is evaluated by testing a 12.5% split of all sensor and insulin pump recordings for each subject, corresponding to 3–12 weeks of data for a subject. The average and the standard deviation of performance indexes are reported in Table 6. The lowest performance indexes was achieved by 2D ConvLSTM models. Bi-LSTM with 1D Convolution layer RNN models achieves the highest accuracy for six subjects out of eleven, and LSTM with 1D Convolution RNN for three subjects. Bi-LSTM with 1D Convolution layer RNN models outperformed other models for 4 subjects with weighted F1 scores ranging from 91.41%−96.26%. Similarly, LSTM models with 1D Convolution layers achieved highest weighted F1 score for another 4 subjects with score values within 93.65%−96.06%. Glycemic events for the rest of 3 subjects showed to be better predicted by regular LSTM models with weighted F1 score between 93.31%−95.18%. This indicates that 1D Convolution improves both the accuracy and F1 scores for most of the subjects. Based on the number of adjustable parameters for the four different RNN models used for a specific subject, LSTMs are the most computational demanding blocks in the model. To assess the computational load of developing the various RNN models, we compared the number of learnable parameters (details provided in Supplementary Materials). These values can be highly informative as the number of dropouts in each model and the number of learnable parameters at each epoch (iteration) is invariant.

A comparison between 1D conv-LSTM and 1D-Bi-LSTM for one randomly selected subject shows that the number of learnable parameters increase by at least 54%, mainly stemming from an extra embedded LSTM in Bidirectional layer (Table S1). While comparing adjustable parameters may not be the most accurate way of determining the computational loads for training the models, they provide a good reference to compare the computational burden of different RNN models.

**Figure 4.**
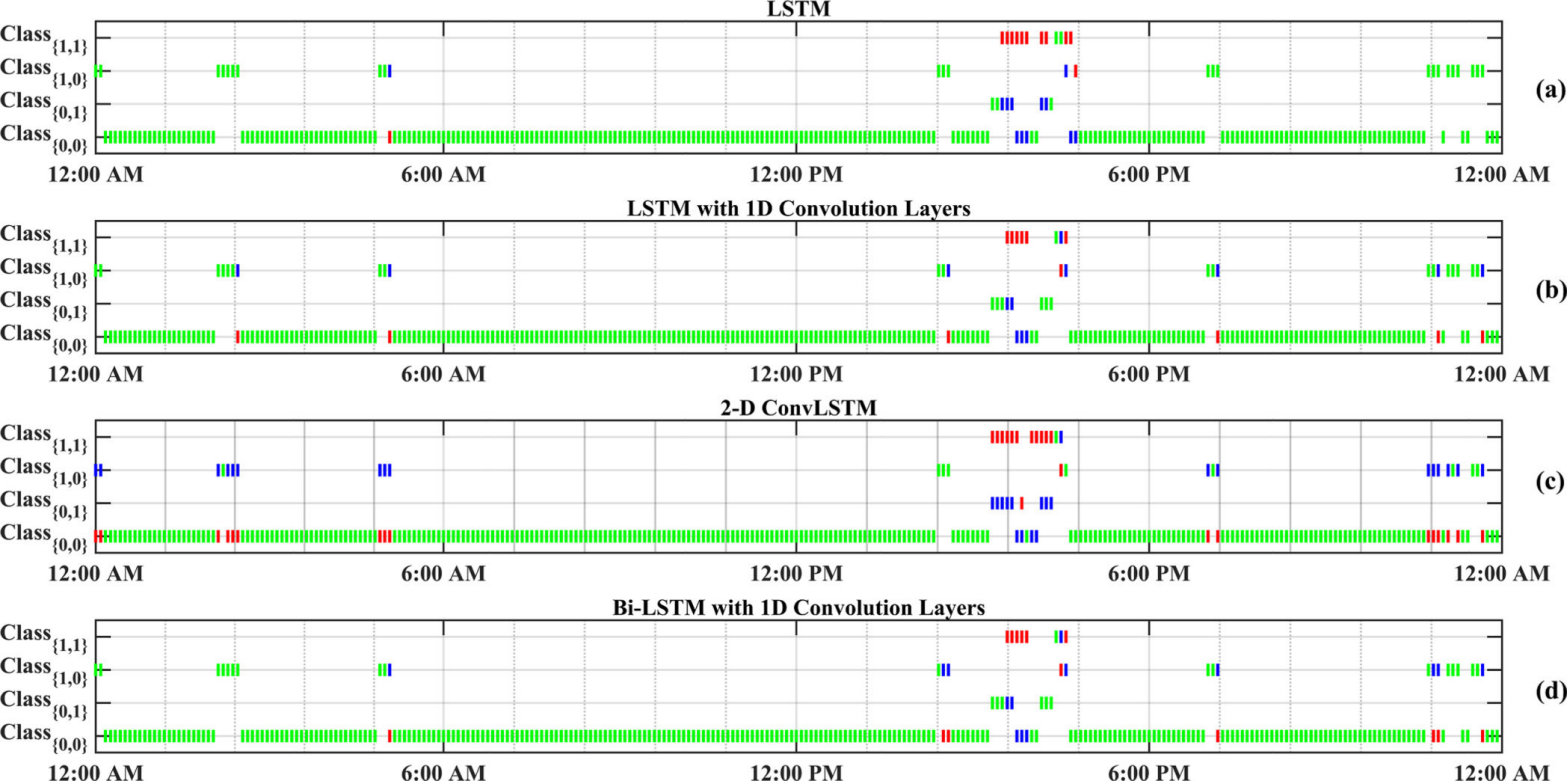
One-step-backward predicted Meal and Exercise events for one randomly selected dataset (Subject 2).Vertical green bars represent correctly predicted classes. Vertical red bars denote incorrectly predicted classes and their actual labels are shown by blue bars. Class Dictionary: *Class*_0,0_: “neither Meal nor Exercise,” *Class*_0,1_: “only Exercise,” *Class*_1,0_: “only Meal,” *Class*_1,1_: “Meal and Exercise”

Figure 4 displays a random day selected from the test data to compare the effectiveness of each RNN model in detecting meal and exercise disturbances. Among four possible realizations for the occurrence of events, detecting joint events, *Class*_1_,_1_, is more challenging as it usually shows overlaps with *Class*_0,1_ and *Class*_1,0_. Another reason for lower detection is the lack of enough information on *Class*_11_, knowing that people usually rather having a small snack before and after exercise sessions over having a rescue carbohydrate during physical activity. Furthermore, the AID systems used by subjects record automatically only CGM and insulin infusion values, and meal and physical activity sessions needed to be manually entered to the device, at times an action that may be forgotten by the subject. Meal consumption and physical activity are two prominent disturbances that disrupt BGC regulation, but their opposite effect on BGC makes the prediction of *Class*_1,1_ less critical than each of meal intake or only physical activity classes.

The confusion matrices of the classification results for one of the subjects (No. 2) are summarized in Table 7. As can be observed from Figure 4 and Table 7, detecting *Class*_0,1_ (physical activity) is more challenging in comparison to carbohydrate intake (*Class*_1,0_) and *Class*_0,0_ (no meal or exercise). One reason for this difficulty is the lack of biosignal information such as 3D accelerometer, blood volume pulse, and heart rate data. Some erroneous detections, such as confusing meals and exercise are dangerous since meals necessitate an insulin bolus while exercise lowers BGC and elimination of insulin infusion and/or increase in target BGC are needed. RNNs with LSTM and 1D convolution layers provide the best overall performance in minimizing such confusions: 2 meals events are classified as exercise (0.003%) and 8 exercise events are classified as meals (0.125%).

The proportion of correctly detected exercise and meal events to all actual exercise and meal events for all subjects reveals that series of convolution-max pooling layers could elicit informative feature maps for classification efficiently. Although augmented features such as the first and second derivatives of CGM and PIC enhance the prediction power of the NN models, the secondary feature maps, extracted from all primary features, shows to be a better fit for this classification problem. Besides, repeated 1D kernel filters in convolution layers suit better the time-series nature of the data as opposed to extracting feature maps by utilizing 2D convolution filters on the data.

## 7. Conclusions

This work focuses on developing RNN models for detection and classification tasks using time series data containing missing and erroneous values. The first modeling issue arose from the quality of the recorded data in free-living. An outlier rejection algorithm was developed based on multivariable statistical analysis, and signal denoising by decomposition of the Hankel matrix of CGM recordings. A multivariate approach based on PPCA for CGM sample imputation was used to keep the harmony and relationship among the variables. The second issue addressed is the detection of events that affect the behavior of dynamic systems and the classification of these events. Four different RNN models were developed to detect meal and exercise events in the daily lives of individuals with T1D. The results indicate that models with 1D convolution layers can classify events better than regular LSTM RNN and 2D ConvLSTM RNN models, with very low confusion of the events that may cause dangerous situations by prompting erroneous interventions such as giving insulin boluses during exercise.

## Supplementary Material

Supplementary Material

## Figures and Tables

**Table 1.** The general demographic information of 11 subjects with T1D and the durations of recorded samples.

Subject	Gender	Age	Duration of Data^1^	Missing Samples (%)	Max Gap Size ^2^
1	M	36	283 days	12.36 %	273
2	M	33	368 days	7.11 %	71
3	F	72	280 days	1.10%	28
4	M	43	468 days	10.03 %	435
5	F	52	655 days	4.91 %	233
6	F	26	206 days	14.45%	107
7	M	51	278 days	6.12 %	34
8	-	41	390 days	8.87%	177
9	-	42	279 days	19.70%	311
10	M	27	695 days	14.32 %	571
11	F	35	413 days	8.97%	147

1 The duration of data is calculated after imputation of missing data and counting gaps between samples.

2 Number of samples, sampling time 5 minutes

**Table 2.** The name and the definition of measured variables.

Variable/Symbol	Definition	Units
CGM	Continuous Glucose Monitoring values sampled every five minutes	mmol/L
Smbg	Self-monitored BGC for sensor calibration	mmol/L
Rate (*Ins_Basal_*)	The basal insulin rate	unit/hr
Bolus(*Ins_Bolus_*)	The actual delivered amount of normal bolus insulin	unit
Time	UTC time stamp	Format: yyyy-mm-yy hh:mm:ss
Duration	The actual duration of a suspend, basal, or dual/square bolus	milliseconds
Activity.name	The type of physical activity	-
Activity.duration (*AD*)	The duration of a physical activity	milliseconds
Distance.value (*DV*)	The value of the distance traveled	miles
Energy.value (*EV*)	The amount of energy spent during activity	kilocalories
Nutrition.carbohydrate (*CHO*)	The carbohydrates entered in a health kit food entry	grams

**Table 3.** The type and definition of the extracted feature variables and the length of time window required for their calculations.

Domain	Feature Description	No. of Required Samples
Time	First derivative calculated by 4*^th^* backward differences	5
	Second derivative calculated by 4*^th^* backward differences	6
Nonlinear	Sign-product of the 1*^st^* and the 2*^nd^* derivatives	6
	Magnitude-product of the 1*^st^* and the 2*^nd^* derivatives	6
Statistical	Statistical measures, namely mean, variance, median, etc., of windowed CGM values	24
	Pair-wise covariance and correlation coefficient between CGM, and its 1*^st^* and 2*^nd^* derivatives	24
Frequency	The magnitudes and frequencies of three dominant peaks in the power spectrum of CGM	288
Model-based	Plasma insulin concentration (PIC) and gut absorption rate (*U_g_*) [40,41].	1

**Table 4.** The description of variables and parameters, and the nominal values of parameters in Hovorka’s model [43].

Variable/Parameter	Description	Value/Unit
$S_{1}(t),S_{2}(t)$	Two-compartment chain representing absorption of subcutaneously administered short-acting insulin	*mU*
Ins(t)	Subcutaneously infused insulin	*mU.min^−1^*
I(t)	Plasma insulin concentration (PIC)	*mU.L^−1^*
$x_{1}(t)$	The remote effect of insulin on glucose distribution	*min^−1^*
$x_{2}(t)$	The remote effect of insulin on glucose disposal	*min^−1^*
$x_{3}(t)$	The remote effect of insulin on endogenous glucose production (EGP)	*min^−1^*
$Q_{1}(t)$	The mass of glucose in accessible compartments	*mmol*
$Q_{2}(t)$	The mass of glucose in non-accessible compartments	*mmol*
$G_{sub}(t)$	Measurable subcutaneous glucose concentration	*mmol.L^−1^*
$U_{G}(t)$	Gut absorption rate	*mmol.min^−1^*
$K_{e}$	The fractional elimination rate of PIC	0.138 *min^−1^*
$k_{a,1}$		0.006 *min^−1^*
$k_{a,2}$	The deactivation rate constants	0.06 *min^−1^*
$k_{a,3}$		0.03 *min*^−1^
$S_{ID}^{f}$	The sensitivity of insulin disposal	0.00082 *L.min*^−1^.*mU*^−1^
$S_{IT}^{f}$	The sensitivity of insulin distribution	0.00512 *L.min*^−1^.*mU*^−1^
$S_{IE}^{f}$	The sensitivity of EGP	0.052 *L.mU*^−1^
$k_{b,1}$		$k_{a,1}\times S_{IT}^{f}$
$k_{b,2}$	The activation rate constants	$k_{a,2}\times S_{ID}^{f}$
$k_{b,3}$		$k_{a,3}\times S_{IE}^{f}$
$EGP_{0}$	EGP extrapolated to	0.0161
	zero insulin concentration	*mmol.kg*^−1^.*min*^−1^
$k_{12}$	The transfer rate constant from the non-accessible to the accessible compartment	0.066 *min*^−1^
τ	The time constant of subcutaneous glucose concentration dynamic	*min*
$V_{g}$	The glucose distribution volume in the accessible compartment	0.16 x *BW(L)*
$V_{I}$	The insulin distribution volume in the accessible compartment	0.12 x *BW(L)*
$F_{R}(t)$	The renal glucose clearance above the glucose threshold of 9*mmol.L*^−1^	$\{\begin{array}{c} 0.003\left(G_{sub}-9\right),G_{sub}\geq 9\\ 0,G_{sub}<9 \end{array}$
$F_{01}$	Non insulin-dependent glucose flux	0.0097 *mmol.kg*^−1^.*min*^−1^
$F_{0,1}^{c}(t)$	The total non-insulin-dependent glucose flux (*mmol.min*^−1^)	$\{\begin{array}{c} F_{01},G_{sub}\geq 4.5\\ F_{01}\left(G_{sub}/4.5\right),G_{sub}<4.5 \end{array}$

**Table 5.** Hardware Specifications.

	Data Preprocessing Task	Model Training Task
CPU Model	Intel i9 9900k	Intel i7 8700k
CPU Frequency	3.6–5.0 GHz	3.7–4.7 GHz
Threads	16	12
RAM Capacity	64GB (DDR IV)	32GB (DDR IV)
Graphics Processor	RTX 2080 Ti × 2	GTX 1050 Ti (GDDR5)
Graphics Memory	11GB	4GB
Clock Frequency	1545–1750 MHz	1290–1392 MHz
Cuda Kernels	4352	768

**Table 6.** The average performance indexes of LSTM, LSTM with 1D convolution layers, 2D ConvLSTM and Bi-LSTM with 1D convolution layers RNN models for the event detection problem. Standard deviations are given in parentheses.

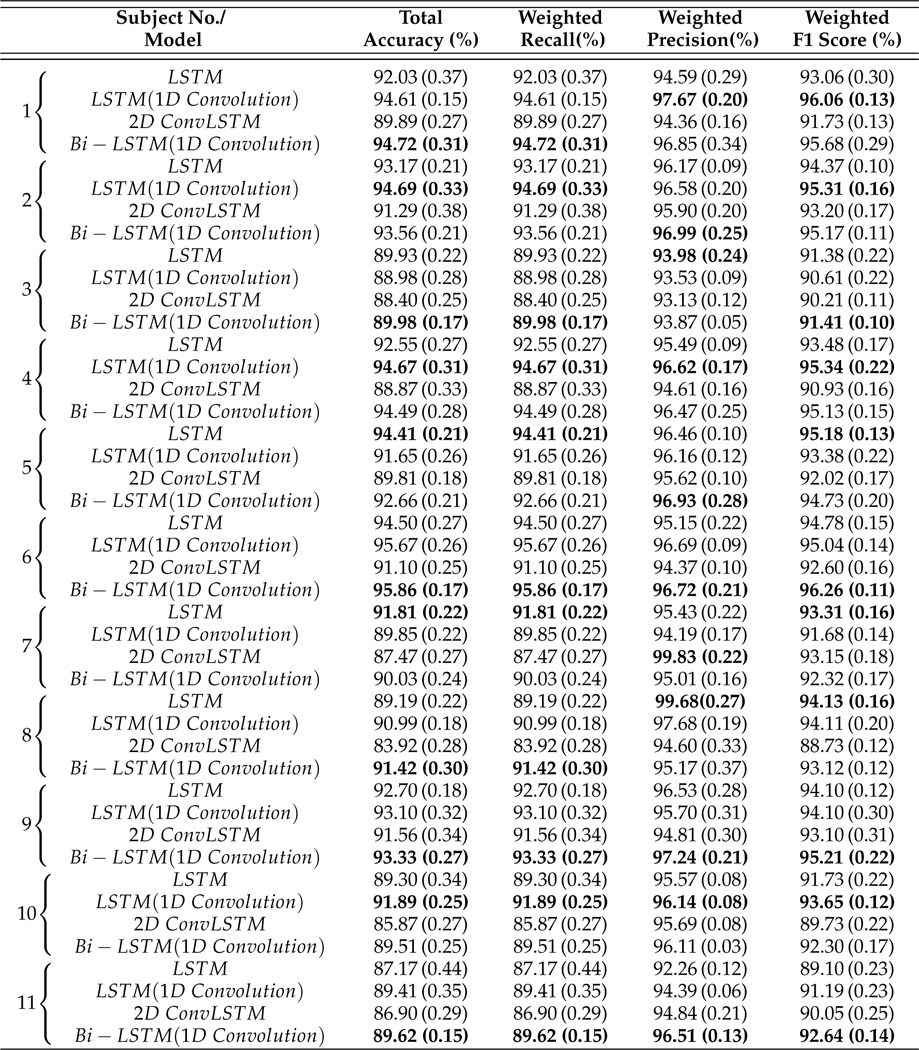

**Table 7.** Confusion matrices calculated from the predicted and actual classes of testing samples collected from Subject 2.