# Supplementary material for: Meal and Physical Activity Detection from Free-living Data for Discovering Disturbance Patterns to Glucose Levels in People with Diabetes
Source: BioMedInformatics. Author manuscript; Available in PMC 2023 Mar 25. (PMC10038808; doi:10.3390/biomedinformatics2020019)
Supplement: Supplementary Material [file NIHMS1871379-supplement-Supplementary_Material.pdf]

SUPPLEMENTARY MATERIALS

Assessing the computational load of training the RNN models

Assessing the computational load of training the RNN models is difficult and can have a large margin of error because of the following reasons:

1- Testing the training time for different models requires providing identical environment for CPU including having all programs closed and having all backend programs and calculations suspended. The first is easy to satisfy but the second is not straightforward in Windows environment. Using commands like "tic toc", is available in both Matlab and Python but is inaccurate in Python. In Matlab environment this command calculates "the number of operations" as well as "the execution time". The execution time is not going to be a reliable because of the second problem stated above, but the "the number of operations" is accurate. Unfortunately, this option is not available in Python environment yet.
2- Backpropagation starts with randomizing all parameters of all NN models and the initial values of these parameters highly affects the execution time. Each time the model is trained, a different time is obtained. Only an approximate value will be obtained by training the model multiple times and averaging the outcomes.
3- Terminating the training of a NN model is performed manually, based on inspecting the learning curve and intuitively deciding when to terminate the training process. This causes a biased interpretation of the execution times.

As a solution to the above issues, we compared the number of learnable parameters. These values can be highly informative as the number of dropouts at each model is fixed and the number of learnable parameters at each epoch (iteration) is invariant. The following table summarizes the number of learnable parameters for each RNN model used in this study for one randomly selected subject (Subject 10).

Table S1. The number of learnable parameters in various RNN models trained in this study.

LSTM Model	Layers	Learnable parameters:
	LSTM	4600
	Dense#1	650
	Drop out #1	0
	Dense#2	650
	Drop out #2	0
	**TOTAL**	**5900**

1D convolutional layer+LSTM	Layers	Learnable parameters:
	Time-distributed 1D convolution #1	2112
	Time-distributed Droup out #1	0
	Time-distributed Maxpool #1	0
	Time-distributed 1D convolution #2	8256
	Time-distributed Droup out #2	0
	Time-distributed Maxpool #2	0
	Time-distributed Flatten	0
	LSTM	11920
	Droup out #3	0
	Dense	420
	Droup out #4	0
	Dense with Softmax	84
	**TOTAL**	**22792**

1D convolutional Bi-LSTM	Layers	Learnable parameters:
	Time-distributed 1D convolution #1	2112
	Time-distributed Droup out #1	0
	Time-distributed Maxpool #1	0
	Time-distributed 1D convolution #2	8256
	Time-distributed Droup out #2	0
	Time-distributed Maxpool #2	0
	Time-distributed Flatten	0
	LSTM	23840
	Droup out #3	0
	Dense	820
	Droup out #4	0
	Dense with Softmax	84
	**TOTAL**	**35112**

Conv2DLSTM	Layers	Learnable parameters:
	ConvLSTM2D	41216
	Droupout #1	0
	Flatten	0
	Time-distributed 1D convolution #2	2580
	Droupout #2	0
	Dense	420
	Droupout #3	0
	Dense with Softmax	84
	**TOTAL**	**44300**

The number of neurons, filters in convolution layers, kernel size (filters in convolution layers) vary from subject to subject. They remain constant for running a model for a subject, with different random seeds.

As a comparison between 1D convolutional LSTM and 1D convolutional Bi-LSTM, technically Bi-LSTM uses one extra LSTM layer in the structure of the model. However, the extra LSTM layer is not added to the model as a sequential layer. This layer is trained simultaneously with the other LSTM layer, but in reverse direction and the outcome of each are merged to obtain the output value. Hence, Bi-LSTM model has more parameters (equal to an extra LSTM layer) to be trained and adjusted.

Based on the above tables, LSTMs are the most computational demanding blocks in the model. A quick comparison between 1D conv-LSTM and 1D-Bi-LSTM, shows that the number of learnable parameters increased by at least 54%, mainly stemmed from an extra embedded LSTM in Bidirectional layer. While comparing adjustable parameters may not be the most accurate way of assessing computational loads required for training models, it provides a good indication about the relative variations in computational loads.
